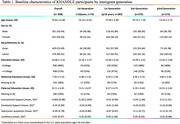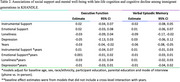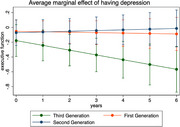# Immigrant generational status, late‐life social support and mental well‐being, and cognitive change in the Kaiser Healthy Aging and Diverse Life Experiences cohort

**DOI:** 10.1002/alz70860_107318

**Published:** 2025-12-23

**Authors:** Joya Deb Lucky, Chelsea Kuiper, Kazi Sabrina Haq, Shelli Vodovozov, Oanh L. Meyer, M. Maria Glymour, Paola Gilsanz, Elizabeth Rose Mayeda, Rachel A. Whitmer, Rachel Peterson

**Affiliations:** ^1^ University of Montana, Missoula, MT, USA; ^2^ Boston University, Boston, MA, USA; ^3^ University of California, Davis School of Medicine, Sacramento, CA, USA; ^4^ Boston University School of Public Health, Boston, MA, USA; ^5^ Kaiser Permanente Northern California Division of Research, Pleasanton, CA, USA; ^6^ UCLA Fielding School of Public Health, University of California, Los Angeles, CA, USA; ^7^ University of California, Davis, Davis, CA, USA

## Abstract

**Background:**

Social support and mental well‐being may differentially provide risk/protection for late‐life cognition among different immigrant generations of U.S. older adults.

**Methods:**

Kaiser Healthy Aging and Diverse Life Experiences (KHANDLE) Asian and Latino participants were categorized as 1st‐generation arriving age <18 (*n* = 73); 1st‐generation arriving age≥18 (*n* = 282); 2nd‐generation (*n* = 279); or ≥3rd‐generation (*n* = 174) immigrants. The NIH Toolbox Emotion Battery assessed emotional support, instrumental support and loneliness. The PROMIS© Depression Instrument assessed depressive symptoms. All were standardized (mean = 0; SD=1) to the U.S. adult population and dichotomized at 0. Verbal episodic memory (VEM) and executive function (EF) were assessed up to 4 times (max. years=6.6) using the Spanish and English Neuropsychological Assessment Scales (SENAS). Linear mixed‐effect models examined associations of social support and mental well‐being with EF or VEM. Separate models tested interactions by immigrant generation. All models adjusted for age, sex, education, race/ethnicity, in‐person vs. phone interview and SENAS language (Spanish vs. English).

**Results:**

Participants’ mean age was 76 years (SD=6.5; Table 1). 1st‐generation immigrants arriving at ages <18 had the lowest instrumental support (mean = ‐0.18, SD=1.0) and highest loneliness (mean = 0.25, SD=0.93) and depressive symptoms (mean = ‐0.04, SD=0.80). In mixed effects models, instrumental support and emotional support were not associated with baseline EF (instrumental β=0.02 [95% CI=‐0.04, 0.07]; emotional β=0.02 [95% CI=‐0.03, 0.08]) or VEM (instrumental β=‐0.02 [95% CI=‐0.08, 0.05]; emotional β=0.01 [95% CI=‐0.06, 0.07]; Table 2). Associations of instrumental support, emotional support, depressive symptoms and loneliness with longitudinal EF and VEM approached null. Compared to generational peers with high instrumental support (ref.) 1st‐generation immigrants with low instrumental support trended toward lower baseline VEM (marginal estimate at the mean (MEM)=‐0.12 [95% CI=‐0.29, 0.04]); 2nd‐generation immigrants with low instrumental support trended toward higher baseline VEM (MEM=0.18 [95% CI=‐0.01, 0.36]); and there was no difference by level of instrumental support among ≥3rd‐generation immigrants (MEM=0.004; 95% CI=‐0.23, 0.24]; immigrant generation*instrumental support *p*‐value=0.05). Among those with high depression symptoms (ref=low depression symptoms), EF decline was only observed among ≥3rd‐generation immigrants (MEM=‐0.07 [95% CI=‐0.11, ‐0.04] Figure 1; depressive symptoms*immigrant generation*years *p*‐value=0.04).

**Conclusion:**

Low instrumental support for 1st‐generation immigrants and high depressive symptoms for ≥3rd‐generation immigrants may be important intervention targets to protect late‐life cognition.